# Understanding ammonia’s role in mitigating concentration polarization in anion-exchange membrane electrodialysis

**DOI:** 10.55730/1300-0527.3703

**Published:** 2024-12-02

**Authors:** Abdallah TIMMAOUI, Mahmoud FERHAT, Nesrine Souad FERHAT, Ahmed HAMDI

**Affiliations:** 1 Mechanics Laboratory, Faculty of Sciences, University of Amar Telidji, Laghouat, Algeria; 2 Laboratory of Physical Chemistry of Materials (LPCM), Faculty of Sciences, University of Amar Telidji, Laghouat, Algeria

**Keywords:** Ion-exchange membrane, mass transport, concentration polarization, electrodialysis, membrane/solution interface

## Abstract

In processes such as electrodialysis, the applied electrical potential is constrained by concentration polarization at the membrane/solution interface. This polarization, which intensifies at higher current densities, impedes ion transport efficiency and may lead to problems such as salt precipitation, membrane degradation, and increased energy consumption. Therefore, understanding concentration polarization is essential for enhancing membrane performance, improving efficiency, and reducing operational costs. This study investigates the impact of ammonia buffer (NH_4_^+^/NH_3_) on sulfate ion transport through anion-exchange membranes with a particular focus on limiting current density and concentration polarization under constant current conditions. The findings demonstrate that ammonia effectively eliminates concentration polarization and enhances chemical reactions at the membrane interface. Notably, the plateau region was absent in the current–voltage curves, as was the transition time in the chronopotentiograms. Furthermore, the Warburg impedance arc in the Nyquist plot of the electrochemical impedance spectra was absent in both limiting and overlimiting current regions and the increasing dominance of the Gerischer arc was registered. At an ammonia concentration of 0.1 M, the influence of concentration polarization on mass transport was effectively mitigated, enabling sulfate counterions to pass through the membrane without encountering concentration polarization. The addition of ammonia catalytically accelerated the proton-transfer reactions, which accelerated the water dissociation reaction at earlier polarization stages, preventing the formation of diffusion boundary layers and facilitating the transport of sulfate counterions through the AMX anion-exchange membrane. As a result, the polarization plateau disappeared and the overlimiting current region shifted closer to the ohmic region, all without affecting the limiting current density (*j_lim_*).

## 1. Introduction

Theoretical and experimental studies on ionic transport phenomena through ion exchangers in the form of membranes have gained popularity and significance in separation processes, desalination, and wastewater treatment industries, in which it is necessary to separate anions from cations, focusing on the separation of anions from cations and allowing the selective passage of one species while excluding the other [1–4]. The ability to selectively target charged substances has made ion-exchange membranes (IEMs) greatly sought after in the fields of separation and water treatment. They are gradually replacing traditional methods such as chemical precipitation, coagulation, activated carbon adsorption, and others [4–6]. IEMs have demonstrated noteworthy benefits in different industrial sectors [6–8]. These membranes offer considerable advantages to society in terms of their impact on the environment and their economic value.

**Figure 1:**
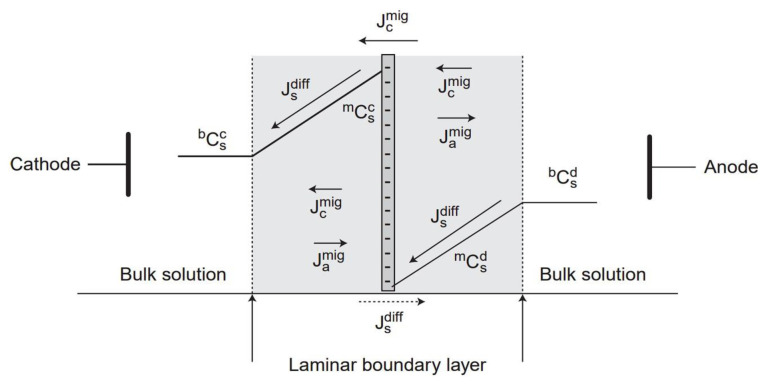
Schematic showing salt concentration profiles and ion flux in a cation-exchange membrane and solutions (adapted from Strathmann [10]).

Electrodialysis is a common membrane process for separating ionic species in solutions [9]. At high current densities, ions and counterions move, creating diffusion boundary layers (DBLs) at membrane/solution interfaces. This causes concentration polarization, forming distinct concentration profiles near the membrane, as depicted in Figure 1 for a cation-exchange membrane.

As current density increases, concentration polarization intensifies, limiting electrical potential. Near the limiting current density (*i_lim_*), membrane resistance rises sharply, typically identified through current–voltage curves (CVCs) [11–14]. Furthermore, concentration polarization catalyzed by the application of an electric field induces alterations in chemical equilibrium, thereby complicating the comprehension of mass transfer phenomena within these systems [15–17].

In practice, if the applied current density exceeds the limiting current value, operational problems occur, such as inorganic salt precipitation on the membrane surface, destruction of the membranes, and an increase in energy consumption, decreasing the process efficiency. To avoid these problems, a current density of about 80% of the limiting current value is generally applied.

Ammonium ions, together with orthophosphoric acid anions and other nutrient-based ions, act as ampholytes in aqueous solutions, meaning that they can undergo protonation or deprotonation reactions that vary with pH. This amphoteric behavior creates unique challenges in ion transport through IEMs, particularly in systems containing these species. Unlike strong electrolytes such as NaCl, KCl, or KNO_3_, which exhibit straightforward transport behaviors, solutions with ampholytes induce complex membrane responses, affecting ion selectivity and stability during processes like electrodialysis. Rybalkina et al. [18] demonstrated that the water-splitting rate at the AMX membrane interface in NH_4_Cl solutions is significantly higher than that in KCl, suggesting that ammonia-containing species facilitate water dissociation by migrating from enriched solutions to the depleted membrane surface. This back-diffusion mechanism plays a critical role in promoting rapid water splitting at the membrane interface. Similarly, Martí-Calatayud et al. [19] documented multiple transition times in chronopotentiometric measurements and identified dual limiting current densities in CVCs during the transport of trivalent metal ions, specifically Cr(III) and Fe(III), through cation-exchange membranes. These findings highlight shifts in the ionic composition in the DBLs and membrane phase, emphasizing the influence of ampholytes on transport dynamics. Furthermore, Kozaderova et al. [20] noted a decline in the flux of ammonium ions at overlimiting current densities, likely due to competitive ion transport and the formation of NH_3_·H_2_O in response to solution alkalization near the cation-exchange membrane. They also observed a similar decrease in nitrate ion flux in overlimiting conditions, attributing this to changes in the functional groups of anion-exchange membranes and their increased catalytic activity toward water dissociation in the presence of ammonium ions. These observations underscore the catalytic impact of ampholytes, such as ammonium ions, on the electrochemical processes within IEMs, contributing to a more complex understanding of transport mechanisms under varying current densities.

To ensure good performance in electrodialysis, it is necessary to better understand the effects of the ampholyte-containing species and protonation/deprotonation reactions on the counterion transport properties during electrodialytic processes, and particularly the effects on concentration polarization and the limiting current. In this study, we use multiple chemical engineering techniques to investigate how the presence of ammonia buffer (NH_4_^+^/NH_3_) affects the transport of sulfate (SO_4_^2−^) counterions through an AMX anion-exchange membrane. The main focus is on the impact it has on the concentration polarization phenomenon and the limiting current density. Our findings provide a deeper understanding of the concentration polarization phenomenon, which can be considered as the key to improving membrane performance, increasing efficiency, and lowering electromembrane processes costs, and enhance our comprehension of the fundamental reaction-transport processes that influence the behavior of ion-exchange systems with solutions containing ampholytes.

## 2. Experimental

### 2.1. Electrochemical cell

**Figure 2:**
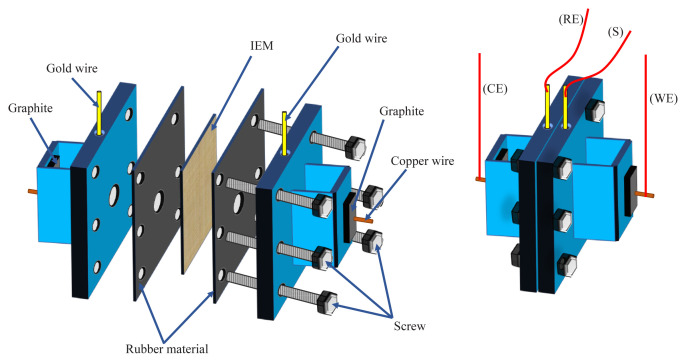
Schematic design of the electrochemical cell.

The electrochemical cell used for the experiments consisted of two symmetrical half cells, each with a volume of 35 mL. An AMX anion-exchange membrane was placed between them. Both compartments contained solutions that had the same concentration and composition. The membrane had an exposed surface area of 0.785 cm^2^. The schematic design of the electrochemical cell is presented in Figure 2. Two gold wire electrodes were used to measure the potential drop across the membrane, while two graphite electrodes were used to control the applied current density. All four electrodes were connected to an SP-150 type potentiostat from BioLogic (Seyssinet-Pariset, France), which was controlled with EC-Lab V10.40 software (BioLogic).

### 2.2. Chemicals

To prepare the solutions, high-quality reagents were dissolved in distilled water. The specific chemicals used included ammonium sulfate ((NH_4_)_2_SO_4_) from Biochem Chemopharma (Cosne-Cours-sur-Loire, France) with purity of 99% and ammonium hydroxide (NH_4_·OH) from Riedel de Haen Chemicals (Buchs, Switzerland) at a concentration of 25%.

### 2.3. Ion-exchange membrane

A Neosepta AMX anion-exchange membrane (Tokuyama Corp., Tokyo, Japan) was used in this study. Its membrane exchange capacity is 1.29 mol/kg swollen membrane and its wet thickness is 0.165 mm. The membrane size is 35 × 50 mm and the exposed surface area is 0.785 cm^2^. The membrane was conditioned based on the NFX 45-200 guidelines set by the French Normalization Association (AFNOR) for IEMs. Following a minimum of 24 h of equilibration in a solution with identical properties to those used in the experiments, the actual experiments were conducted at room temperature without stirring the solutions.

### 2.4. Current-voltage curves

CVCs were recorded by measuring the relationship between current and voltage using a constant current scanning rate of 10 μA/s to determine the limiting current density. The point at which the derivative curve changed direction within the specific current range was used to estimate the maximum current density.

### 2.5. Chronopotentiometry and electrochemical impedance measurements

Chronopotentiometry and impedance measurements were performed using the same equipment. The measurements were taken at specific working points on the polarization curves, which represented the three different regions of the CVCs (ohmic, limiting, and overlimiting regions).

In electrochemical impedance spectroscopy (EIS), a system is subjected to both a sinusoidal current and a direct current (DC). The experimental frequency range employed in the present study spanned from 5 kHz to 3 mHz, with current amplitude set at 60 μA. These specific frequency and amplitude values were chosen by reviewing numerous other studies in the literature and conducting a number of experiments [18,21–23].

## 3. Results and discussion

### 3.1. Current–voltage characteristics

#### 3.1.1. CVC of AMX anion-exchange membrane in ammonium sulfates

**Figure 3:**
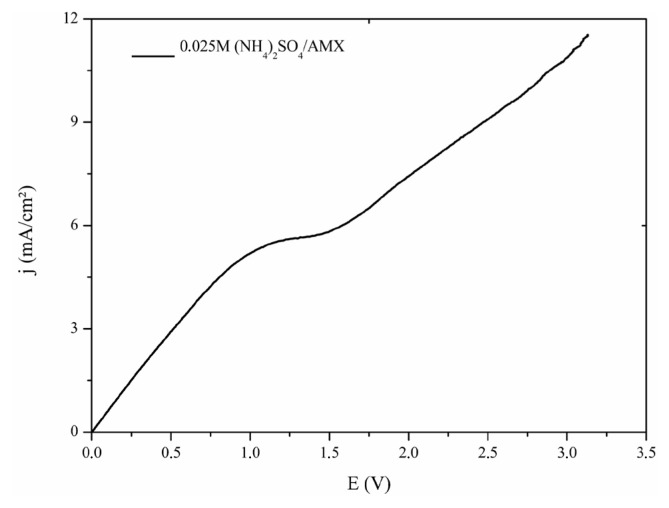
CVC of the AMX membrane in 0.025 M (NH_4_)_2_SO_4_.

Figure 3 shows a typical CVC obtained for the AMX membrane in 0.025 M ammonium sulfate solution ((NH_4_)_2_SO_4_). As can be seen, the obtained curves are typical for a monopolar anion-exchange membrane, with three distinct regions identified [24–27]. Initially, a linear relationship between the current and the voltage drop was registered, referred to as the ohmic region. In this phase, the applied electric field drives counterion migration through the anion-exchange membrane, causing ion enrichment and depletion in the adjacent compartments. In the ohmic region, the system adheres to Ohm’s law, exhibiting a direct linear correlation between the applied voltage and the resulting current [4,26]. However, as the current density increases, the influence of concentration polarization becomes more significant. This leads to a decrease in concentration at the thin boundary layer between the membrane and solution, resulting in an increase in resistance [12,13]. As a consequence, the curve deviates from linearity. When the current density reaches the limiting value (*j_lim_*), the counterion concentration in the depleted solution approaches zero, which results in the appearance of the plateau at the limiting current, indicating the onset of the polarization region. Beyond this plateau, the current density resumes its increase again, which is referred to as the overlimiting region in the CVC. For decades, the underlying mechanisms driving this increase were not well understood. However, in this region, additional ion transport mechanisms, such as electroconvection and water dissociation, become prominent, ensuring the current transport [16,28].

#### 3.1.2. Effect of ammonia on the CVC of AMX membrane in ammonium sulfate solution

**Figure 4:**
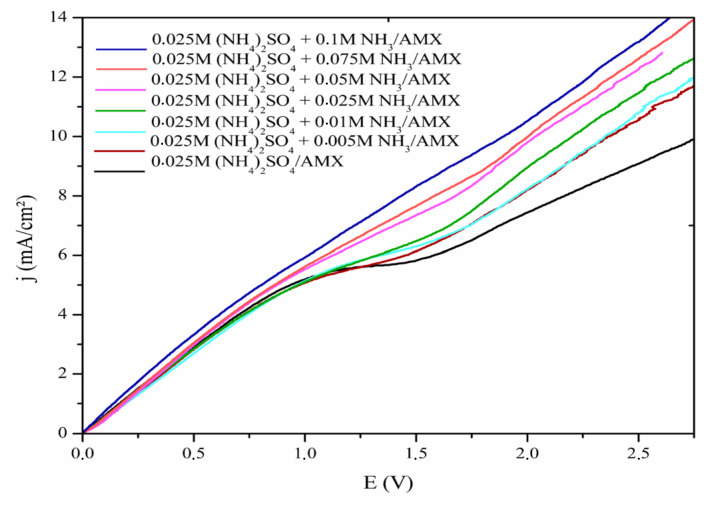
CVCs of AMX membrane in 0.025 M ammonium sulfate solution with different ammonia concentrations.

To examine the effect of ammonium/ammonia buffer (NH_4_^+^/NH_3_) on the transport of sulfate counterions (SO_4_^2−^) through the AMX anion-exchange membrane, we progressively added ammonia (NH_3_) from 0.005 M to 0.1 M to the AMX membrane/0.025 M (NH_4_)_2_SO_4_ system. The results are presented in Figure 4, where the CVCs of the system without ammonia are presented in black and other colors are used for systems with different NH_3_ concentrations.

It is evident that as the concentration of ammonia increases, the length of the polarization plateau in the limiting current region decreases and its slope becomes steeper. As a result, the polarization plateau is nearly nonexistent when the ammonia concentration is 0.075 M. Additionally, when the ammonia concentration reaches 0.1 M, the polarization plateau is eliminated (blue curve in Figure 4). In this scenario, the sulfate counterions can be transported through the membrane without experiencing concentration polarization. However, the introduction of ammonia eliminates the polarization plateau without influencing the value of limiting current density *j_lim_*.

In the limiting current region, the transport of sulfate counterions through the AMX anion-exchange membrane is expected to be constrained by the development of a DBL at the membrane/solution interface, resulting from the concentration polarization phenomenon. However, it can be inferred that the addition of ammonia acted catalytically, preventing the formation of the DBL at the membrane/solution interface. This facilitated the transport of sulfate counterions through the membrane, eliminating the polarization plateau and shifting the overlimiting current regions toward the ohmic regions.

In accordance with classical concentration polarization theory and in line with the electroconvection theory presented by Rubenstein and colleagues [29–31], it seems unlikely to observe a decrease or elimination of the concentration polarization plateau under these circumstances.

### 3.2. Electrochemical impedance spectroscopy measurements

#### 3.2.1. EIS of AMX membrane in ammonium sulfate solution at different current values

EIS is a valuable technique in studying complex systems with multiple layers and complicated surfaces [32,33]. In the field of membranes, it is used to characterize surface fouling [23] and to study ion transfer kinetics across different types of IEMs [11].

The impedance response of IEM systems commonly presents at least two contributions to ion transport, which are described by two distinctive arcs in Nyquist diagrams [11,34–37]. The first arc is a geometric arc that occurs at high-frequency values (10^5^ to 10^3^ Hz) and is associated with the geometric capacitance and ohmic resistances of the membrane and its adjoining regions, the bulk solution, and the Luggin capillaries. The second one is a diffusion arc known as a Warburg-type arc, appearing at the right low frequencies with a finite length associated with the transport of ions by diffusional mechanisms. This arc follows a 45° slope at medium frequencies and evolves into a semicircle as the frequency tends toward zero. A third arc of Gerischer impedance can also be observed at intermediate frequency values. This arc typically occurs when chemical reactions take place at the membrane/solution interface [18,37,38].

Depending on factors such as the flow regime, ion concentration, and polarization level, each contribution may vary in its significance concerning the overall system impedance.

To better understand the effect of ammonia buffer on mass transport, we conducted EIS measurements at different current bias levels in this investigation. These levels were specifically selected to cover the three different CVC regions (ohmic, limiting, and overlimiting region).

To ensure the reliability and reproducibility of our results, each experiment was performed in triplicate, with error bars displayed in the figures to illustrate measurement precision. Error bars serve as critical indicators of data variability, where larger error bars suggest greater dispersion potentially due to instrumental sensitivity, environmental fluctuations, or sample instability, while smaller error bars indicate high repeatability and measurement precision, underscoring the robustness of the findings. These trends highlight the importance of experimental consistency, as repeated trials better capture true variability. Thus, the patterns in error bars provide valuable insights into the stability and reliability of each dataset, which is essential for validating our findings and interpreting implications for electrodialysis and ion-exchange system transport properties.

For our EIS data, the error bars show marked variability, especially at lower frequencies. This phenomenon is typical in EIS measurements due to increased susceptibility to external noise and system fluctuations in this range. However, as measurements approached higher frequencies or higher concentrations, the variability tended to stabilize, evidenced by smaller error bars. This pattern was likely a result of the system reaching a steady state or an optimal condition where external factors exerted less influence.

**Figure 5:**
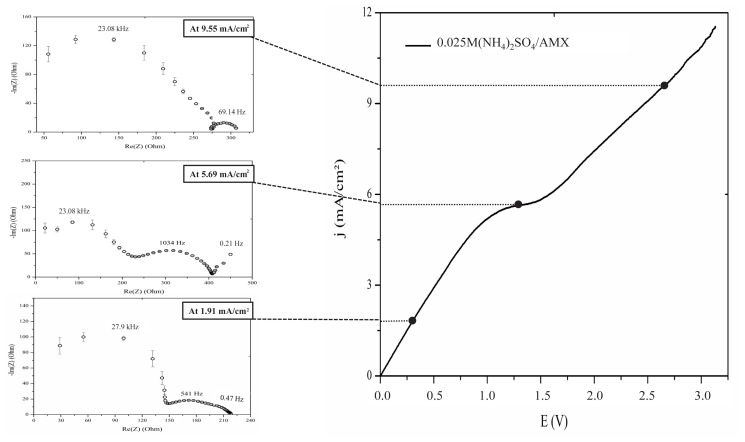
CVC and EIS spectra of AMX membrane in 0.025 M ammonium sulfate solution at different current densities.

Figure 5 presents the obtained EIS spectra for the AMX membrane in 0.025 M (NH_4_)_2_SO_4_ solution at different current densities. The scaling of the real part (Re(Z)) and the imaginary part (−Im(Z)) of the impedance axis was adjusted to enhance the clarity of each Nyquist plot.

In the plot corresponding to the quasi-ohmic region (lower left panel) and at lower current densities, the geometric and diffusive arcs associated with the geometric capacitance and ohmic resistances of the system are registered at 27.9 kHz and 0.47 Hz, respectively. Additionally, the majority of the system resistance is attributed to the geometric semicircle, whereas the Warburg-type diffusion impedance, which reflects the transformation of concentration variations in the quasi-electroneutral solution adjacent to the membrane into changes in the potential drop, is significantly smaller. These results are consistent with the lower level of polarization achieved at 1.91 mA/cm^2^, indicating that the concentration profiles may not be fully developed; in other words, the concentration of the counterions may not have reached a value close to zero at the membrane surface. Thus, in this regime, the limitations caused by diffusion are not significant yet, and so the main resistance to the transportation of ions is associated with their migration.

The existence of the Gerischer arc at frequencies around 541 Hz indicates the occurrence of chemical reactions near the surface of the membrane. In our case, this can be explained by the involvement of secondary and tertiary amines, found in the fixed groups of the AMX anion-exchange membrane, in proton-transfer reactions with water [37].

At limiting current density *j_lim_* (middle left panel), the membrane’s resistance, determined from the intercept with the x-axis at lower frequencies [36], increases compared to the resistance in the ohmic region. This increase in resistance was anticipated because of concentration polarization near the membrane’s surface. In this case, the concentration profiles at the membrane/solution interface must be fully developed. Accordingly, the electrolyte concentration at the diluate membrane/solution interface tends toward zero and a highly resistant DBL is formed, such that the main resistance to ion transport is located in this part of the system where ion transport becomes limited by diffusion processes rather than the intrinsic electrical conductivity of the membrane.

In addition, the Gerischer arc’s size expanded and its characteristic frequency shifted from 541 Hz to 1034 Hz. The Gerischer impedance reflects the effective resistance of the chemical reaction occurring at the membrane/solution interface, with its value corresponding to the chord length of the Gerischer arc. The characteristic frequency of this impedance, marked by the peak of the arc, is associated with the effective rate constant of the water dissociation reaction. This constant represents the rate-determining step for a chemical reaction under an electric current in an electrode or membrane system. In this case, the dissociation reaction produces protons (H^+^) and hydroxyl (OH^−^) ions. For anion-exchange membranes, the characteristic rate constant typically exceeds 1000 s^−1^, corresponding to a frequency in the range of several hundred hertz [18,21]. This arc, often observed in the middle frequency region of the impedance spectrum, is characteristic of both bipolar [39] and asymmetric membranes.

A key parameter obtained from each semicircle is the characteristic frequency, defined as the measurement point at which the semicircle reaches its maximum. The inverse of this frequency represents the time constant for the specific process captured by the semicircle, providing insights into the rate at which ion transport processes occur until the membrane system achieves a new steady state [36]. Thus, the shift of the characteristic frequency to a higher value indicates that the proton-transfer reactions with water were improved by increasing the current density [40].

It is noteworthy that the Warburg-type impedance at low frequencies had a distribution that resembled semiinfinite diffusion [41]. For porous electrodes, this is due to the fact that charge carriers are unable to fully penetrate the material layer, which leads to difficulties in obtaining a fully developed system response across the entire probed surface with low-frequency signals [42,43]. Thus, based on the experimental findings reported by Pismenskaya et al. [44] and Martí-Calatayud et al. [36], it can be concluded that concentration gradients are not well established within the DBL and the length of these layers is not clearly defined. Therefore, the incomplete development of the DBL is caused by the catalytic dissociation of water and the transportation of OH^−^ ions through the membrane.

In the EIS spectra of current density values above *j_lim_* (upper left panel), the membrane’s overall resistance is lower than the resistance at the polarization region. Moreover, the frequency associated with the second arc on the right is approximately 69.14 Hz, which is significantly different from the frequencies associated with Warburg-type impedance.

In anion-exchange membrane electrodialysis, the pH of the internal solution in the membrane consistently exceeds that of the bathing solution because H^+^ ions are excluded from the membrane [18,37,44]. This means that when a polybasic acid reaches equilibrium with the membrane, the ion composition in the membrane is enriched with doubly and triply charged acid anions compared to the external solution. Consequently, when entering the membrane, some of the singly charged anions become multicharged anions and the liberated H^+^ ions return to the depleted solution. In our case, the proton transfer reactions occurring between water molecules and ammonium ions can be represented by the following equations:


(1) $${{NH}_{4}}^{+}+{OH}^{-}⇌{NH}_{3}+H_{2}O$$

(2) $${NH}_{3}+H_{2}O⇌{{NH}_{4}}^{+}+{OH}^{-}$$

In the literature, it is reported that the Gerischer arc can be subdivided into two distinct subarcs in specific systems [37,40,44]. The first subarc, seen at higher frequencies, is associated with the dissociation of water. The second subarc, which occurs at lower frequencies, is linked to slower homogeneous reactions happening in the electrolyte. Therefore, the arc observed at 69.1 Hz can be attributed to the reaction between ammonium ions and water molecules as shown in Eq. (1). These reactions have a slower rate compared to water splitting and are therefore observed at lower frequencies.

#### 3.2.2. Effect of ammonia concentration on EIS of AMX membrane in ammonium sulfate solution

**Figure 6:**
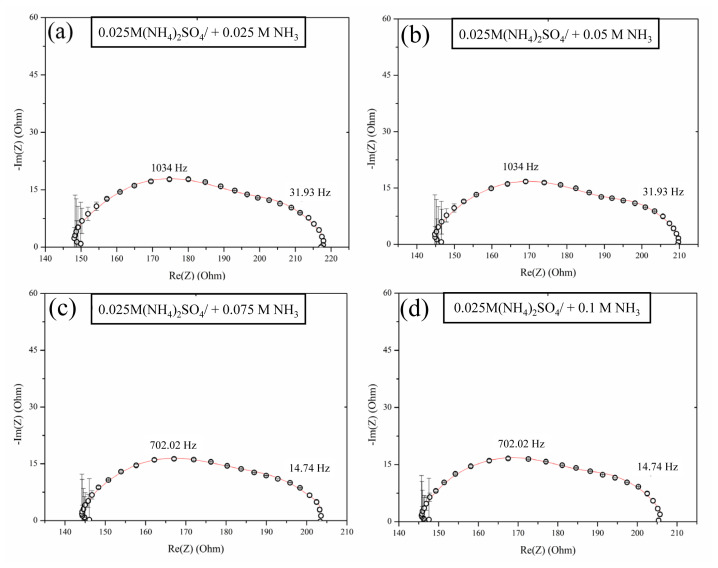
EIS spectra of AMX membrane in 0.025 M (NH_4_)_2_SO_4_ solution with different NH_3_ concentrations in the ohmic region (*j_ohm_* = 1.91 mA/cm^2^).

**Figure 7:**
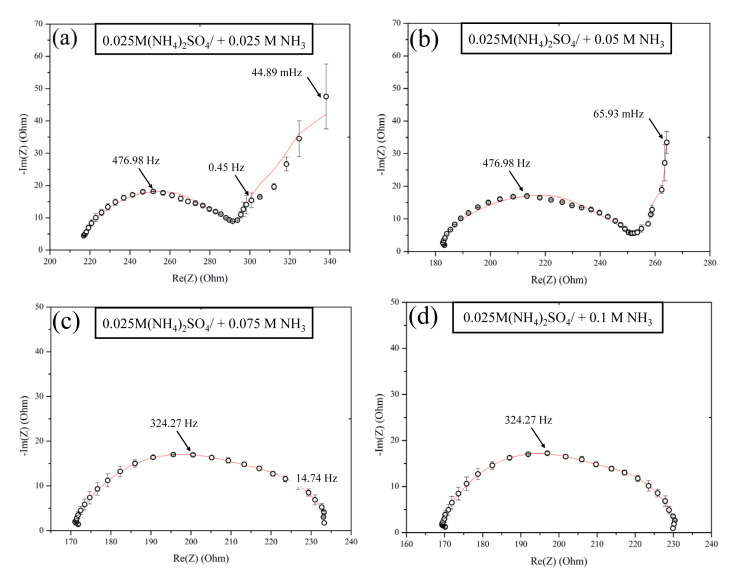
EIS spectra of AMX membrane in 0.025 M (NH_4_)_2_SO_4_ solution with different NH_3_ concentrations in the polarization region (*j_lim_* = 5.69 mA/cm^2^).

**Figure 8:**
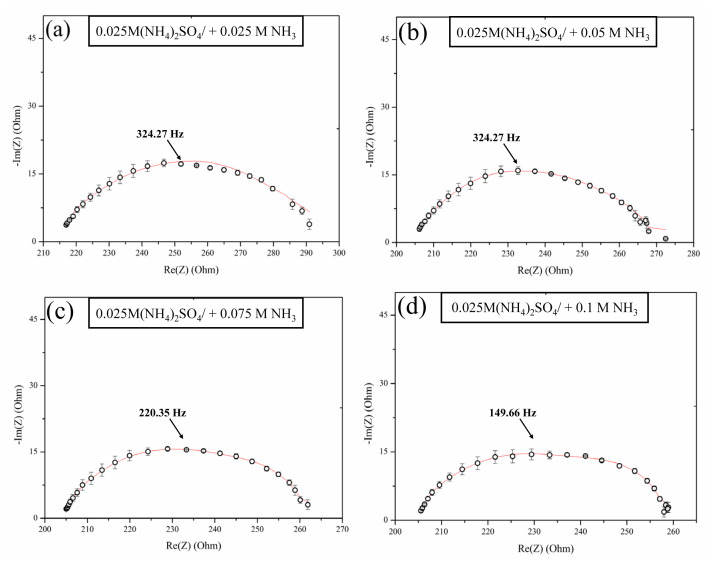
EIS spectra of AMX membrane in 0.025 M (NH_4_)_2_SO_4_ solution with different NH_3_ concentrations in the overlimiting current region (*j_over_* = 9.55 mA/cm^2^).

The impedance spectra of the AMX anion-exchange membrane in 0.025 M (NH_4_)_2_SO_4_ solution with increasing NH_3_ concentrations are shown in Figures 6, 7, and 8. The geometric arc was not observed at higher frequencies (130–10 kHz) in the Nyquist plots across all spectra. In the ohmic region (Figure 6), the spectrum showed two overlapping arcs regardless of the NH_3_ concentrations. Similar to cases where no ammonia was added, the Gerischer arc was observed, indicating chemical reactions. The frequency of this arc was significantly higher when NH_3_ was present compared to systems without ammonia. Therefore, it can be assumed that the NH_3_ enhanced the proton-transfer reactions with water molecules involving the secondary and tertiary amines in the fixed groups of the AMX membrane. Furthermore, the Gerischer arc contributed predominantly to the system’s overall resistance, suggesting that these reactions are the main mechanism at play in this case.

There were significant changes in the impedance spectrum shape in the polarization region (Figure 7). When the ammonia concentration was less than 0.075 M, a low-frequency Warburg impedance arc was present, indicating semiinfinite diffusion. This arc contributed significantly to the system’s overall resistance, suggesting that ion migration is the dominant mechanism in this setup. However, while increasing the ammonia concentration, the impedance spectra no longer showed the low-frequency arc and the Gerischer arc became the main contributor to system resistance. This change points to water dissociation as the dominant mechanism at higher ammonia levels. The presence of the Warburg impedance arc is attributed to the conversion of changes in concentration in the quasi-electroneutral solution next to the membrane into changes in potential drop. Once the limiting current is reached, the concentration profiles should be fully established. The existence of Warburg impedance with semiinfinite diffusion in our system, where the ammonia concentration is less than 0.075 M, indicates that the concentration gradients are not well developed throughout the DBL. Therefore, the elimination of the Warburg impedance by increasing ammonia concentrations indicated that the effect of the DBL was negated by the addition of ammonia even in the limiting current regime. These findings are consistent with the counterion transport shown in the CVCs without a concentration polarization plateau.

The EIS spectra obtained at 9.55 mA/cm^2^, a current density value above the *j_lim_* value (Figure 8), indicate that regardless of the NH_3_ concentration, the impedance spectra show that all arcs in the Nyquist plots merge into one arc within the Gerischer impedance frequency range. Comparable findings were documented by Kniaginicheva et al. [21] and Martí-Calatayud et al. [40], who examined the dissociation of water in IEMs and discovered that the arcs merge at current densities greater than 1.5. *j_lim_*. In our study, the merging of arcs in the Gerischer impedance region suggests that water dissociation is the predominant mechanism driving ion transfer under overlimiting conditions. This shift in mechanism is likely due to the enhanced role of proton-transfer reactions facilitated by the presence of ammonia, which accelerates dissociation processes at the membrane interface. This arc convergence and the increasing dominance of the Gerischer arc further suggest that water dissociation is responsible for maintaining ion transport beyond the typical concentration polarization limits, potentially reducing the impact of diffusion limitations on overall membrane resistance.

It is worth noting that when the concentration of NH_3_ increases, the overall resistance of the membrane decreases. This decrease in resistance is attributable to the catalytic dissociation of water at the membrane/solution interface and the transfer of OH^−^ ions through the membrane.

### 3.3. Effect of ammonia on the chronopotentiograms of AMX membrane in ammonium sulfate solution

**Figure 9:**
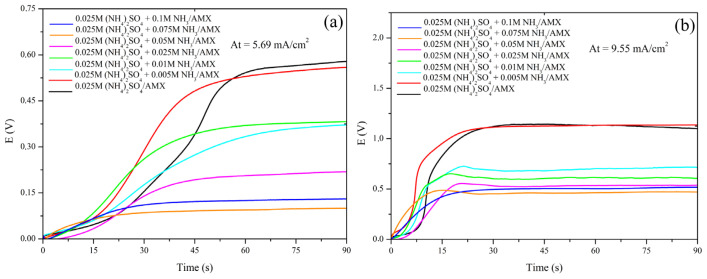
Chronopotentiograms of AMX membrane in 0.025 M (NH_4_)_2_SO_4_ solutions with different NH_3_ concentrations in the (a) limiting and (b) overlimiting current regions.

Chronopotentiometry was utilized to validate the findings described above. The results shown in Figure 9 illustrate the changes in voltage over time in both the limiting and overlimiting current regions. In the limiting current region (Figure 9a), the curve of ammonium sulfates without ammonia displays a noticeable change in direction that corresponds to the transition time of the system. Furthermore, as the concentration of ammonia increases, the significance of the transition time in the curves decreases until it completely disappears at an ammonia concentration of 0.75 M. This provides physical evidence that the addition of ammonia eliminated the effect of the DBL on sulfate counterion transport, even in the limiting current region. A similar response in terms of chronopotentiometry was seen in the overlimiting region (Figure 9b). The transition time could be clearly observed only when ammonium sulfates were used without ammonia. Moreover, it was somewhat visible with lower concentrations of ammonia, but it completely disappeared as the concentration of ammonia increased. Eventually, at a concentration of 0.75 M, the transition time was completely eliminated, indicating that the effect of the DBL on mass transport at the membrane/solution interface had been completely negated and concentration polarization was eliminated. These findings are consistent with the CVCs, which showed a decrease, and elimination of the polarization plateau in the limiting current region as the concentration of ammonia increased.

It is important to note that there was a decrease in the potential difference across the membrane in the chronopotentiograms after it reached its highest point. This decrease in potential difference is usually attributed to gravitational convection and/or water dissociation reaction [26]. Thus, in this specific case, the decrease of the potential difference at a higher ammonia concentration is associated with the fact that the ammonia chemically enhanced the water dissociation reaction at the membrane/solution interface.

### 3.4. Hypothesis of catalytic enhancement of chemical reactions at the membrane/solution interface

Ammonia played the role of a water dissociation facilitator at the membrane/solution interface. With its neutral electrical charge, it could easily diffuse through the DBL, where the water dissociation reaction took place. By reacting with protons that were excluded from the membrane surface and those coming from water molecules (Eq. (2)), ammonia allowed for the passage of hydroxyl ions (OH^−^) through the AMX membrane. In a phenomenon similar to facilitated diffusion or facilitated electrodiffusion, explaining the electrostatic interactions of ions at the surface and in the pores of the membranes, where the transport of neutral amino acids was enhanced across the H^+^ type cation-exchange membrane and the OH^−^ type anion-exchange membrane, the NH_4_^+^ present in the electrolyte reacts with OH^−^ ions present inside the AMX membrane and those coming from the water dissociation reaction, producing the uncharged NH_3_ species. For “facilitated” diffusion, this phenomenon is possible due to the shift of the pH of the internal solution of the AEM to the alkaline region compared to the external solution as a result of the Donnan exclusion of H^+^ ions from the membrane as co-ions. Hence, some of the NH_4_^+^ ions lost their charges and were transformed into NH_3_ molecules (Eq. (1)), which were not excluded from the membrane. These NH_3_ species then diffused across the membrane to the depleted solution side, where they converted back into NH_4_^+^ ions again and released OH^−^ ions, which returned to the enriched membrane side. This process increased the concentration of NH_3_ molecules on the depleted side of the membrane, allowing for reactions that enhanced water dissociation and affected the onset potential of the overlimiting region, shifting it toward lower values and reducing the length of the polarization plateau.

In a system without ammonia, the applied electrical potential causes water molecules to dissociate. When ammonia is present, it lowers the energy needed for this process, especially as the ammonia concentration increases. With the applied electrical potential, neutral ammonia, and the membrane’s charged groups, water molecules dissociate faster than usual. This speeds up the overlimiting current transport by adding more hydroxyl ions near the membrane/solution interface beyond the accumulation layer of sulfate ions. It also boosts the movement of negative charges, like those of sulfates and hydroxyl ions, through the membrane and reduces concentration polarization.

## 4. Conclusion

The results obtained in this study showed that ammonia addition significantly influenced ion transport behavior, particularly within the concentration polarization and overlimiting current regions. As the ammonia concentration increased, CVC analysis showed a pronounced decrease in the polarization plateau length and a steeper slope. At 0.075 M NH_3_, the plateau was reduced, and at 0.1 M, it vanished entirely, as the NH_3_ suppressed the concentration polarization effect by preventing the development of the DBL at the membrane/solution interface, thus facilitating sulfate ion transport without impacting the limiting current density.

In the impedance spectra, a low-frequency Warburg arc indicating semiinfinite diffusion was present at lower ammonia concentrations, but as the ammonia concentration increased, this arc faded and the Gerischer arc dominated, showing that water dissociation became the primary mechanism and implying that water dissociation dominated under conditions of higher ammonia. Moreover, under overlimiting conditions, the Gerischer arc consistently merged with others, indicating that water dissociation, facilitated by NH_3_, was the primary ion transport mechanism. The enhanced water dissociation and OH^−^ transfer reduced the overall membrane resistance, emphasizing the role of NH_3_ in optimizing ion transport by mitigating diffusion limitations and concentration polarization.

By clarifying these fundamental processes, our findings not only enhance the academic understanding of the processes but also provide guidance for optimizing ion-exchange systems for various applications, including desalination, separation processes, and wastewater treatment.
